# Mutual dosing of tungsten, molybdenum and selenium impact anaerobic digestion microbiome

**DOI:** 10.1007/s10534-026-00790-1

**Published:** 2026-01-28

**Authors:** Kris Anthony Silveira, Javier Ramiro-Garcia, Cian Lawless, Jose Manuel Espinosa-Vazquez, Fernando G. Fermoso, Gavin Collins, Vincent O’Flaherty

**Affiliations:** 1 Microbial Ecology Laboratory, School of Biological and Chemical Sciences and Ryan Institute, University of Galway, University Road, Galway, H91 TK33 Ireland; 2 Bioprocess for the Circular Economy Group, Consejo Superior de Investigaciones Cientificas (CSIC), Instituto de la Grasa, Campus Universitario Pablo de Olavide-Ed. 46, Ctra. De Utrera, Km 1, 41013 Seville, Spain; 3 Microbial Communities Laboratory, School of Biological and Chemical Sciences, University of Galway, University Road, Galway, H91 TK33 Ireland

**Keywords:** Anaerobic digestion, Microbiome, Molybdenum, Selenium, Tungsten, Trace metal dosing, Methanogenic granules

## Abstract

Metals are critical in anaerobic digestion, but their co-occurrence effects on microbiome structure and function are underexplored. This study hypothesized that exposure of methanogenic granules to a trace element (TE) mixture alongside molybdenum (Mo), tungsten (W) or selenium (Se)-would alter (i) extracellular polymeric substances (EPS) protein and carbohydrate content, (ii) microbial composition and function (iii) methanogenic pathways.

To test this, anaerobic batch reactors (n = 35) were set up in a fed batch mode, with sacrificial reactors (n = 14) used to collect biomass for analyses, including DNA: RNA co-extraction, amplicon sequencing, and determination of the concentrations of total and soluble metals, Scanning Electron Microscopy- Energy Dispersive X-ray (SEM–EDX) and EPS extraction over a 24-day period.

The results reveal that, Mo and W increased the concentration of soluble Fe in abiotic controls, enhancing Fe and S retention. The presence of W, Mo, W + Se, and Se had a positive effect on methane production, with W + Se and W enhancing acetoclastic methanogenesis. Additionally, Se increased EPS protein and carbohydrate contents in the biomass. Shifts in the microbiome composition were mainly driven by Mo and Se, with typically dominant *Anaerolineacaeae*, *Capriciproducens, Macelibacteroides* and *Clostridium *sensu stricto* 5* taxa. Functional potential suggested an enrichment of nucleotide metabolism and, importantly, Vitamin (B12, B6 and B9) metabolic potential.

These finding inform Anaerobic digestion (AD) stakeholders about the impacts of Fe, W, Mo, and Se co-dosing on process performance and microbiome structure and function, offering insights to optimize biogas production through tailored metal supplementation combinations, given demonstrations at lab and pilot scales.

**Supplementary Information:**

The online version contains supplementary material available at 10.1007/s10534-026-00790-1.

## Introduction

The increased implementation of anaerobic biotechnologies for the treatment of organic wastes provides pathways towards sustainable environmental management and underpins the UN’s Sustainable Development Goals, particularly those relating to clean water and sanitation, and affordable and clean energy (Zhu et al. 2022). Anaerobic digestion (AD) provides wastewater treatment and resource recovery. The power of such biotechnologies is the microbial biomass (sludge). Granular sludge is composed of individual self-aggregating and retentive methanogenic granules. Indeed, high-rate granular bioreactors are popular due to their capacity to treat a variety of high strength/ toxic wastewaters, complemented by high sludge retention within the bioreactor. Despite years of implementation, a key knowledge gap remains in the mechanistic understanding of sludge granulation and associated AD process improvements. Unravelling these fascinating biofilms is key to unlocking their capacity for resilience (Mills et al. 2023).

AD functions are supported by four microbial guilds—hydrolysers, acidogens, acetogens, and methanogens (Weinrich and Nelles 2021). Each of these guilds require macro- and micronutrients, involved in the maintenance of homeostatic function. Populations within each trophic group consistently require essential – e.g., iron (Fe), nickel (Ni) and cobalt (Co) – and trace elements, e.g., tungsten (W), molybdenum (Mo) and selenium (Se) for energy conservation, biosynthesis, and cellular maintenance (Choong et al. 2016; Garuti et al. 2018). The necessity of Fe, Ni and Co in AD systems has been heavily scrutinised, largely where industrial feedstocks are concerned (Glass and Orphan 2012; Zhu et al. 2022). While their interactions have been shown as essential for AD, as deficiency and overload have been linked to process decline, microbiome destabilisation and operational problems (Šafarič et al. 2020).

W, Mo and Se are routinely included in micronutrient mixtures for standard biomethane potential (BMP) tests (Angelidaki et al. 2009), synthetic (Worm et al. 2009) and industrial (Ali et al. 2022) feedstock. However, the mechanisms affected by metal supplementation have not been delineated in complex AD microbiomes. Rather, studies on metal addition have been constrained to pure and co-cultures (Chen et al. 2016; Li et al. 2023).

Literature establishes broad trace element (TE) dosing requirements for a variety of industrial feedstocks. However, these vary significantly with reactor scale and configuration, source and type of sludge, initial TE concentration, and substrate characteristics. Insufficient TE level within feedstock or sludge biomass led to a decline in process performance, necessitating TE addition. (Weinrich and Nelles 2021).

Mo, W and Se have been used for specific purposes in AD before. For instance, Se has shown to stabilise performance in bioreactors under high ammonia stress treating chicken manure (Molaey et al. 2018), in thermophilic (Yirong et al. 2015) and mesophilic AD (Šafarič et al. 2020) with high concentrations of volatile fatty acids (VFA). Se addition increased nanoparticle precipitates of selenide causing higher total solids/ volatile solids (TS/VS) and settling velocity (Logan et al. 2023). Additionally, acetogens are known to respire using Se cofactors as terminal electron acceptor (Tan et al. 2016) through the Wood-Lunghdahl pathway, which support antioxidant defence mechanisms (Staicu and Barton 2021). Additional Mo or Se resulted in 30–40% higher methane production from food waste (FW), even when combined with a metal mixture (Facchin et al. 2013). Mo also prevents the formation of toxic sulphides which can precipitate and deplete the microbiome of TE (Nemati et al. 2001). W and Mo act as enzymatic cores in metal based pterins, transcriptional regulators and regulators of FeMo cofactor biosynthesis (Aguilar-Barajas et al. 2011; Iobbi-Nivol and Leimkühler 2012; Hille et al. 2016). W has been shown to improve biogas production efficiency from food waste (Das and Mondal 2013), alleviate VFA accumulation (Šafarič et al. 2020) and enhance methanogenic activity in TE-deficient systems (Arthur et al. 2022).

Since Mo, W and Se are central to anaerobic metabolism, Se-dependent enzymes require W for full functionality and SeO_3_^−^ could interfere with Fe and S metabolism that impact Mo/W incorporation (Fermoso et al. 2015). Thus, presenting a unique biogeochemical interaction and potential synergy. It is imperative to understand the outcomes of such a co-occurrence in bioreactors. Additionally, as W and Se are mechanistically distinct i.e. W impacts redox enzymes, while Se impacts EPS, protein synthesis and stress protection. Therefore, a combined metabolic and structural co-operation is highly probable, such an approach has not been examined before.

This study explores how simultaneous addition of a TE mix and either Mo, W, W + Se or Se influences methanogenic granules – precisely in terms of (i) specific methanogenic activity rate (ii) extracellular polymeric substances (EPS) protein, carbohydrate levels, and (iii) changes in microbial composition and potential function. The goal is to understand how abiotic interactions of W, Mo and Se influences Fe, Ni, Co, Cu solubility and retention, and record synergistic or antagonistic impacts on the biotic components of AD such as methanogenic pathways, EPS production, and the overall microbiome.

## Materials and methods

### Source of biomass

Anaerobic (methanogenic) sludge granules were sourced from a full-scale bioreactor (NVP Energy, Ireland) treating skimmed dairy milk wastewater at ambient temperatures (8–25 °C). The sludge was stored at 4 °C under N_2_-flushed conditions, and was characterised by determining TS/VS, concentration of all metals from sludge, and pH.

### Fed-batch incubations with Mo, W and Se exposure

**Fig. 1:**
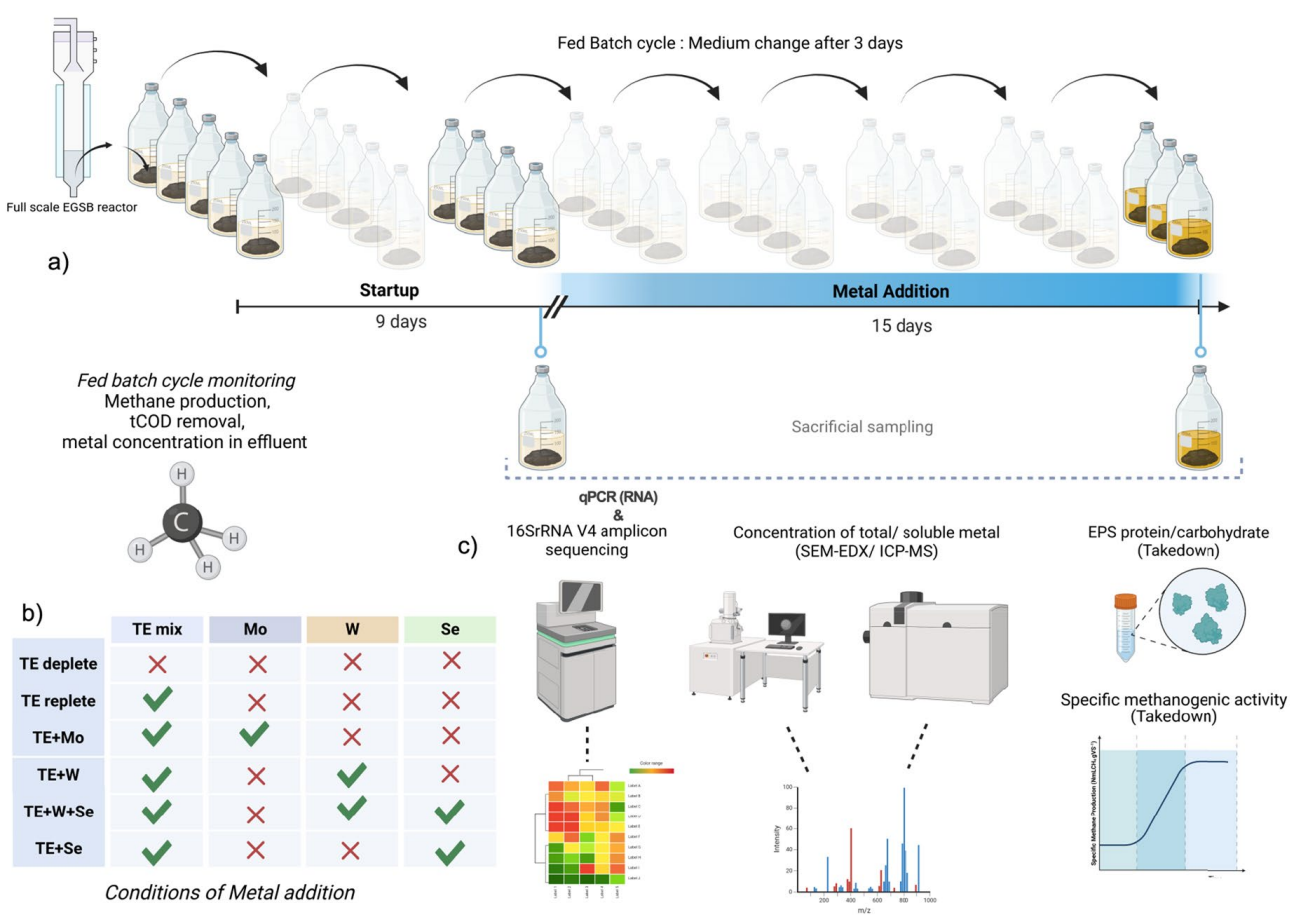
Schematic illustration of the experimental layout for the fed batch trial. **a** Representation of the fed-batch cycles. **b** Conditions applied to the anaerobic bioreactors during metal addition/ test phase. **c** Suite of techniques performed on sacrificially sampled biomass. This image was prepared in Biorender

Preliminary experimental validation found that maximal methane production took place within three days, this was set as the incubation cycles residence time. Nitric acid bath (10%) was used to decontaminate all glassware and materials used in the experiment. Total forty-two anaerobic bioreactors were set up in 250 ml serum bottles, which were used to assess the effect of metal exposure on methanogenic granules. Reactors were separated into six conditions (Fig. 1b and Table 1), each involved five test reactors and two abiotic controls. An abiotic control had fresh medium without biomass, it served as negative control, determination of true concentration of soluble TE available and blanks for tCOD removal calculations.

**Table 1:** Concentrations of metals during the metal addition phase of the trial (day 9 to 24) for each group of anaerobic reactors. Selected concentrations are average dosing values recommended for optimum AD process as per (Fermoso et al. 2015; Weinrich and Nelles 2021)

Groups	Trace element mix				Metals		
	Fe(µM)	Co(µM)	Ni(µM)	Cu(µM)	Mo(µM)	W(µM)	Se(µM)
*Control conditions*							
TE deplete	–	–	–	–	–	–	–
TE replete	158	77	39	37	–	–	–
*Test conditions*							
TE + Mo	158	77	39	37	2	–	–
TE + W	158	77	39	37	–	1.7	–
TE + W + Se	158	77	39	37	–	1.7	2.7
TE + Se	158	77	39	37	–	–	2.7

Synthetic medium containing (gCOD l^−1^): lactose (3.6), ethanol (5.1), propionate (0.5) and butyrate (0.6) was prepared according to Mills et al. (2021). The buffer was modified from NaHCO_3_ to MOPS (3-(N-morpholino) propane sulfonic acid) (10 mM) due to non-interactions with metal ions (Ferreira et al. 2015).

The bioreactors were operated in fed-batch mode, with a cycle consisting of three stages; ‘fill’, ‘react’ and ‘decant’. The Fill stage involved the addition of 150 ml fresh medium (9.8 gCOD l^−1^), respective trace metal solutions (Table 1) to aliquoted biomass (20 gVS l^−1^), reaching a substrate-to-inoculum ratio of ~ 1:2. The bottles were then sealed with butyl rubber stoppers, crimped with aluminium caps, and the headspace was exchanged five times with N_2_ gas.

Next, the react stage comprised continuous stirring 150 rpm at 37 °C. Headspace pressure (Keller LEO1 digital manometer) and biogas content were monitored regularly. After pressure readings, reactors were vented and pressure re-set values noted, thus obtaining a pressure difference. Methane volume was quantified from the pressure differences, using the Ideal gas law equation; then converted to standard temperature and pressure. For each batch methane yield was calculated from total methane volume generated per gram carbon (gCOD).

The decant stage involved overnight settling and removal of effluent medium. The effluent was used to determine pH, metal concentration, and total chemical oxygen demand (tCOD).

In total, eight fed-batch cycles were performed. Batches 1–3 (Days 0–9) were considered the start-up period, where only synthetic medium was added. This facilitated acclimatisation of biomass to synthetic medium and environmental conditions. Batches 4–8 (Days 9–24) represented the test period, when the effect of respective TE, and co-occurrences, were determined (Fig. 1).

Sacrificial sampling of one representative reactor from each condition was performed by taking down the reactor: then sampling and storing this biomass for EPS extraction, total metal extraction, DNA/ RNA co-extraction, and SEM–EDX analysis. Sacrificial samples were saved from batch 3 (Day 9; before metal exposure), and batch 8 (Day 24; after metal exposure).

### Analytical methods

The methane content of biogas was measured in triplicate on a Gas chromatograph (GC; Varian) utilizing a flame ionization detector with Nitrogen as a carrier gas at a flow rate of 25 ml min^−1^; 3 ml gas sampled from reactor headspace using syringes. COD was quantified using a kit (Reagcon, Shannon, Ireland) (0 – 1500 mg l^−1^ range), following the manufacturer’s instructions, and using a Hach DR3900 Laboratory VIS Spectrophotometer at OD 435 nm. MOPS increased COD measurement therefore the COD of abiotic controls were subtracted from test reactor. A portable (Thermo Scientific™ Eutech™ pH 150 Meter) was used to measure influent and effluent pH. APHA recommended loss on ignition technique used to estimate TS/VS of sludge samples (Telliard 2001) before and after metal exposure.

### EPS extraction

EPS was extracted using a gentle heating method according to Li and Yang (2007). Briefly, 5 g wet biomass (~ 0.2 gVS) was centrifuged at 3000 g for 15 min, and the supernatant was discarded. The biomass was suspended in 0.05% NaCl solution, immersed in a 70 °C water bath for 30 min, centrifuged at 20,000 × g at 4 °C for 30 min and the supernatant was collected. The protein and carbohydrate content of the EPS were measured from the supernatant using Bichonic Acid assay and Anthrone method with standard curves generated using bovine serum albumin (BSA) and glucose, respectively (Hasani Zadeh et al. 2022). The samples were measured on a UV spectrophotometer (BioRad iMark Microplate reader, USA) at 562 and 620 nm. Concentrations were expressed as mg of glucose equivalent l^−1^ of carbohydrates and mg of BSA equivalent l^−1^ of protein, then normalised to gVS biomass.

### Metal quantification

Concentration of soluble metals from effluent was obtained by filtration (0.22 µm filter) and acidification (~ 5% nitric acid). The filtrate was subjected to ICP-OES (inductively coupled plasma – optical emission spectroscopy) 5110 synchronous vertical dual view (ICP-OES 5110, Agilent 7 Technologies, Santa Clara, USA). (Operating parameters: viewing mode: radial; viewing height: 8 mm; radiofrequency power: 1.20 9 kW; plasma gas: 12 l min^−1^; nebulizer flow: 0.70 l min^−1^).

Solid metals were extracted from sludge by microwave assisted digestion (CEM MARS 5 Microwave Accelerated Reaction System, North Carolina, USA; US EPA Method 3051A, 2001). The aqua regia extracted fraction was then filtered with a 0.22 µm filter and subjected to inductively couple plasma–Mass spectrometry (Operating conditions: viewing mode: radial; viewing height: 10 mm; radiofrequency power: 1600 W; plasma gas: 15 l min^−1^; nebulizer flow: 0.61 l min^−1^). Certified reference material sludge (ERM®—CC144) was used as a positive control to validate extraction efficiency.

For SEM–EDX, the biomass was stored in a sodium cacodylate and glutaraldehyde-based electron microscopy fixative for 2 h. The fixed biomass was then passed through an ethanol dehydration series (30, 50, 70, 90 and 100%). After air drying, samples were suspended in a paraffin embedding resin and sliced using cryostat microtome (Leica CM3050 S, Wetzler, Germany), to expose the internal surfaces of the spherical granules. The slices were mounted onto poly –L- lysine coated glass slides and secured using double sided carbon tabs. Samples were then sputter-coated with gold (Emitech K550, East Sussex, UK). Imaging was performed using a scanning electron microscope (SEM Hitachi S4700, Tokyo, Japan) at an acceleration voltage of 15 kV and at 50 µA current. Energy dispersive X-ray (EDX) spectroscopy coupled with SEM was used for elemental analysis of granular sludge biomass. For each condition, 9–12 sections were subjected to EDX analysis; this provided EDX spectrums from which abundance (mass fraction) of metals were calculated.

### Specific methanogenic activity

Specific activity of methanogenic pathways in the biomass exposed to metal dosing was measured. For this, sodium acetate (30 mM), methanol (30 mM) or H_2_:CO_2_ (80:20 v/v; 1.5 atm) was added to triplicate sets of 150 ml serum vials each containing 5 g wet biomass (~ 1 g VSl^−1^) and basic anaerobic medium as prepared by Collins et al. (2003) to quantify amount of methane produced. The maximum slope of exponential change in methane production was used to determine the rate of methanogenesis.

### Nucleic acid extraction and sequencing

Triplicate sludge samples (~ 5 ml) were sampled from the bioreactors on day 9, and day 24: the liquid fraction was discarded, and approximately 250 mg (wet weight) of biomass was added to sterile, bead-beating tubes (Macherey–Nagel Bead Tubes Type A, Germany), flash-frozen in liquid nitrogen and stored at −80 °C. DNA and RNA was co-extracted from the biomass following a phenol–chloroform-isoamyl alcohol (25:24:1) protocol (Griffiths et al. 2000), incorporating a minor modification in the lysis buffer composition to include 4% polyvinylpyrrolidone (PVP), which facilitates removal of humic acids and phenolics (Schrader et al. 2012). DNA and RNA quality and quantity were determined using a Nanodrop spectrophotometer (Thermo Fischer Scientific), 1% (w/v) agarose gel electrophoresis, and Qubit dsDNA BR and ssRNA BR assay kits (Thermo Fischer Scientific). Samples were normalised to (50 ng µl^−1^) and sent for sequencing of the V4 region of 16S rRNA gene amplicons using the 515F and 806R primer pair (Caporaso et al. 2010) on an Ilumina NovaSeq 6000 platform (Novogene, Cambridge, UK).

To investigate the active prokaryotic community, aliquots of the DNA/RNA co-extracts were treated with DNase (NZYtech, Lisbon, Portugal), following the manufacturer’s instructions. DNase-treated samples were then cleaned using an NZY total RNA isolation kit, the absence of genomic DNA was validated by end-point PCR. cDNA was synthesized from purified RNA using a Luna Script cDNA synthesis kit (NEB, MA, USA) with No Reverse transcription controls, following the manufacturer’s guidelines. Active bacterial community abundance was measured using qPCR assays with primers from Maeda et al. (2003). Controls and standards were included as recommended by MIQE guidelines (Bustin et al. 2009).

### Bioinformatics and statistical analysis

Sequencing data analysis was carried out using a validated pipeline for 16SrRNA amplicon analysis called NG-Tax, with default parameters (Ramiro-Garcia et al. 2018). Taxonomy was assigned using SILVA 138 SSURef database (Quast et al. 2012). Alpha and beta diversity as measured using R package phyloseq 1.44.0 (McMurdie and Holmes 2013) and picante 1.8.2 (Kembel et al. 2010). Plots were generated using ggplot 2-3-5-1 (Wickham 2016) package. Putative functional analysis from assigned ASV were identified by PICRUSt 2 (Douglas et al. 2020). Differential analysis was performed using MicrobiotaProcess 1.13.1.992 package (Xu et al. 2023).

Statistical analysis of the fed batch trial data sets was performed in Microsoft Excel. For this, a one-way ANOVA (α = 0.05 or otherwise mentioned), with Tukey honestly significant difference (HSD) post hoc test to categorise pairwise differences. All data points are provided as mean of triplicate measurements ± standard deviation or represented by error bars. Correlation was calculated using Pearson’s correlation coefficient and tested using one tailed t-test.

## Results

### TE addition positively associated with methane production

The theoretical methane production from the carbon substrates provided to each bioreactor (1.5 gCOD), calculated through conserved stoichiometry and the Buswell equation, was 0.51 l (Zulkifli et al. 2019). The actual production of methane by the bioreactors was in the range 0.23–0.43 1 i.e., 61–86% of the theoretical methane yield. The tCOD removal efficiency of the bioreactors was ~ 90%, implying low concentration of particulate matter (suspended biomass) in the aqueous phase and less interference with the measurement of COD; therefore, tCOD is used as a metric of carbon conversion and steady state condition (Fig. S2).

**Fig. 2:**
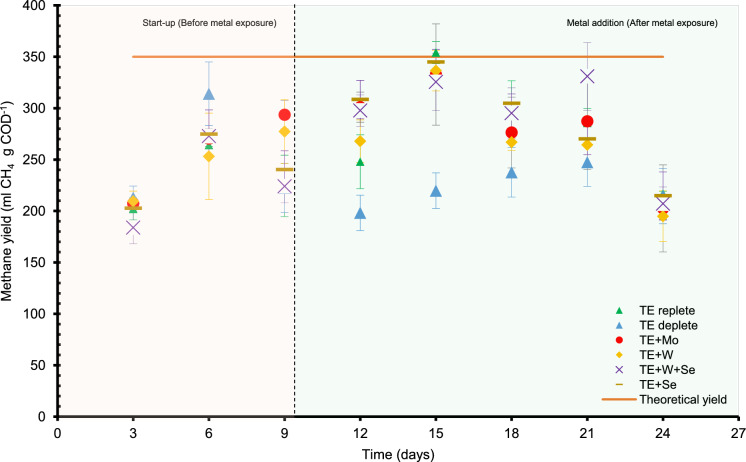
**a** Methane yield from anaerobic reactors after React stage of each batch. The dashed line indicates start of metal exposure

Methane yield by day 15 (maximum methane production from the batch) was significantly (p < 0.05) (10–36%) higher in TE-replete bioreactors than in TE-deplete incubations. Furthermore, in the case of TE + Se (p < 0.05) (33 – 17%) a higher rate than in TE-deplete conditions were noted, while revealing no other significant differences between groups (Fig. 2).

All the groups had a methane yield of more than 50% after start up. Additionally, endpoint pH after each fed batch cycle was ~ 6.8 to 7.1 for all conditions. Both methane yield and pH indicated stable AD system.

### W with Se increased methanogenic rate from acetate

Acetate methanogenic rates were significantly higher (p > 0.01) when exposed to TE + W + Se, even higher in TE + W (p > 0.05) and TE + Se groups. Methanogenic rates from H_2_: CO_2_ were highest in the TE + W group, compared to all other groups (Table 2). Methanogenesis from methanol as substrate demonstrated a longer lag phase of more than three days (data not provided), while the inoculum had the highest (p > 0.01) methanogenic rate compared to all the tested conditions.

**Table 2:** Specific methanogenic activity (mlCH_4_ gVS^−1^ day^−1^) from acetate (30 mM), H_2_: CO_2_ (80:20 (v/v), 1.5 atm), or methanol (30 mM). Inoculum from full scale reactor. Values are based on triplicate bottles and data provided as mean ± standard deviation

Methanogenic pathway	Conditions						
	Inoculum	TE-deplete	TE-replete	TE + Mo	TE + W	TE + W + Se	TE + Se
Acetate	218 ± 7	183 ± 12	207 ± 7	200 ± 32	187 ± 7**	232 ± 21*	203 ± 5
H_2_: CO_2_ (80:20%)	28 ± 7	27 ± 16	33 ± 1	40 ± 6	49 ± 6	47 ± 15	37 ± 5
Methanol	25 ± 6*	2 ± 0	5 ± 1	6 ± 1	3 ± 0	2.41 ± 1	5 ± 1

*indicates statistically significant (p < 0.01) difference between; Acetate (TE + W + Se and TE-deplete) and Methanol (Inoculum vs all groups) **indicates statistically significant (p < 0.05) difference between the groups tested. Acetate (TE + W and TE + W + Se)

### Mo and W affected retained Fe concentrations

#### Soluble metals in abiotic controls.

The concentration of soluble metals to which the biomass was exposed, was determined by subtracting the metal concentrations from abiotic controls of TE-replete vs other groups (n = 2). Effective concentrations during exposure were 1.7 – 1.9 µM W, 1.3 – 1.5 µM Se, 1.9 µM Mo, 63.3 µM Co, 36.1 µM Ni and 27.6 µM Cu at each fed-batch cycle in metal addition phase.

Markedly, the presence of W and Mo resulted in higher concentrations of soluble Fe (from 58—61 µM to 134 µM) when both metals co-occurred in the abiotic control. The soluble metals present in the reactors were successfully removed. However, removal % for Fe and Cu reduced across batches (Fig. S4).

### Fe retention in the presence of Mo, W, and W + Se

**Fig. 3:**
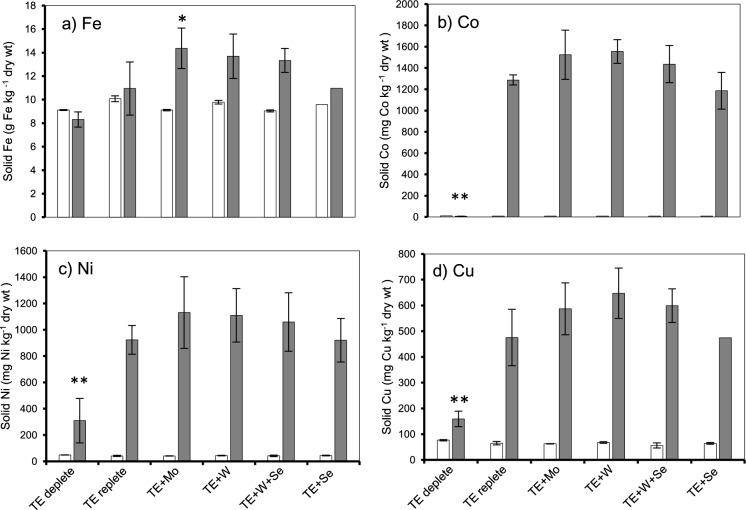
Solid metal concentration of **a** Fe, **b** Co, **c** Ni, and **d** Cu expressed as g or mg metal kg^−1^ dry weight biomass. Measure obtained during two time points; before metal exposure (white bar, day 9) (n = 3) and after metal exposure (grey bar, day 24) (n = 5); (*) indicates statistically significant difference (p < 0.05) between TE + Mo and TE-deplete groups, (**) indicates statistically significant difference (p < 0.05) between TE-deplete and all groups

Prior to exposure, the concentration of Fe and Co were 9 – 10 g Fe kg ^−1^ dwt and 8–10 mg Co kg ^−1^ dwt, respectively (Fig. 3a). Moreover, under Mo, W, and W + Se dosing, retained Fe increased from 10.9 to > 13.3 g Fe kg ^−1^ dwt.

Here, co-dosing caused a substantial increase in total Co concentration compared to TE-deplete conditions. Co concentration increased when W was present (1481 mg Co kg ^−1^ dwt), compared to TE replete group (1292 mg Co kg ^−1^ dwt) (Fig. 3b). Comparing all conditions, the concentration of Fe, Ni, Co, Cu and S were same on day 9 but follow a related retention trend on day 24 (higher for TE + Mo and TE + W groups).

Cu and Ni accumulated in the TE-deplete group, from 65 and 41 after start up to 159 and 309 after takedown respectively (Fig. 3c and d).

The co-occurrence of W and Mo also slightly increased Cu and Ni concentrations; within TE + W group, Cu increased from 475 to 647 mg Cu kg ^−1^ dwt and in TE + Mo group from 475 to 586 mg Cu kg ^−1^ dwt. while within TE + W group, Ni increased from 923 to 1109 mg Ni kg ^−1^ dwt and in TE + Mo group from 923 to 1130 mg Ni kg ^−1^ dwt (Fig. 3c and d).

**Fig. 4:**
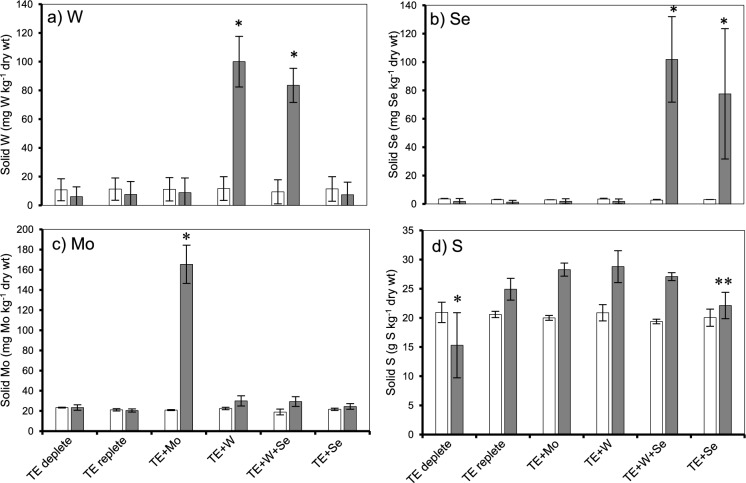
Solid metal concentration of **a** W **b** Se **c** Mo and **d** S expressed as g/ mg metal kg ^−1^ dry weight biomass measured at two time points. Before metal exposure (white bar, day 9) (n = 3) and after metal exposure (grey bar, day 24) (n = 5); (*) indicates statistically significant difference (p < 0.05) between TE-deplete and all groups, (**) indicates statistically significant difference (p < 0.05) between TE + Se and TE + Mo, TE + W

We observed the presence of Mo, W, Se and S metals, meanwhile verifying the absence of these metals in other groups (Fig. 4). On the co-occurrence of W and Se, W was retained at a higher concentration when present on its own (100 mg W kg ^−1^ dwt), compared to when added with Se (83 mg W kg ^−1^ dwt) (Fig. 4a). Despite no direct S addition, elevated levels in TE + Mo, TE + W and TE + W + Se group (Fig. 4d) likely originating from S-containing feed compounds or degradation products of organic matter.

#### SEM–EDX based validation of trace metal exposed biomass

The measurement of metals in solid, soluble fraction, and SEM–EDX spectrums indicated that the added metals were present within dosing concentration and retained by the biomass through physico-chemical and microbial processes.

**Fig. 5:**
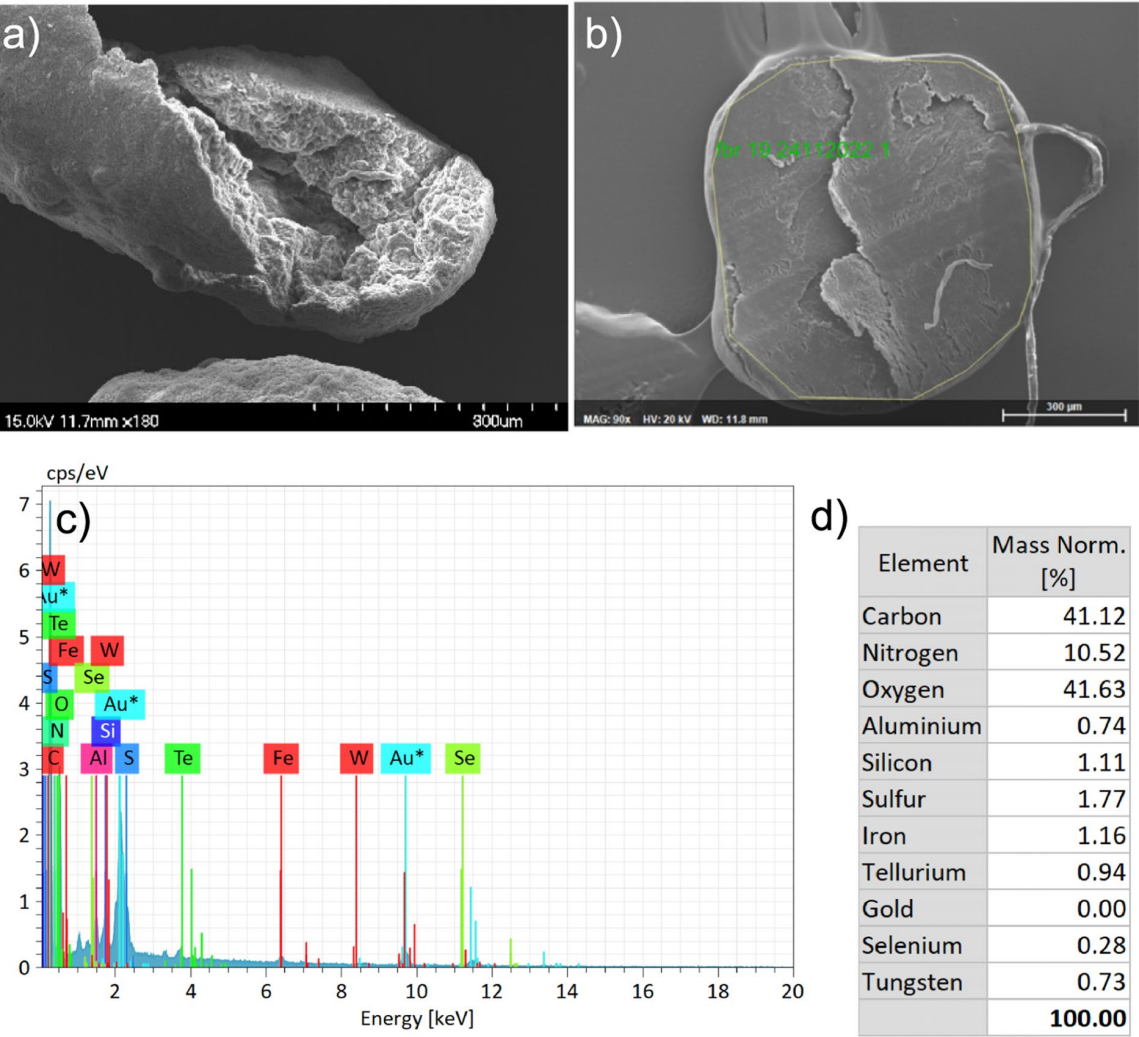
Representative images from TE + W + Se fixed **a** whole granule surface and **b** sliced granule sections. **c** Energy dispersive X-ray spectra of the presence of W and Se, spectral cps/eV counts are converted to **d** normalised mass fractions for the targeted area

W occurred at a higher frequency across slices of granular sludge (in 78% sections) exposed to W (n = 9) and W + Se (n = 12), when compared to TE-replete, TE-deplete and control EDX spectra (Fig. 5). The Fe mass fraction increased slightly when W (1.4), W + Se (1.5) and Se (1.5) compared to granule sections from TE-replete incubations (1.2). Sulphur (S) was found to be least abundant when Se was present. Se was observed in EDX spectra, but its occurrence and mass fractions were not significant. Rather, only (Tellurium) Te was observed to be present, and increased in mass fraction, when Se was present.

### Se dosing affected EPS protein and carbohydrate content of methanogenic granules

Se (1.9 mg) induced a significantly higher production of EPS protein (105 mg BSA equivalent gVS^−1^) and carbohydrates (2 mg glucose equivalent gVS^−1^) versus all the groups tested.

**Fig. 6:**
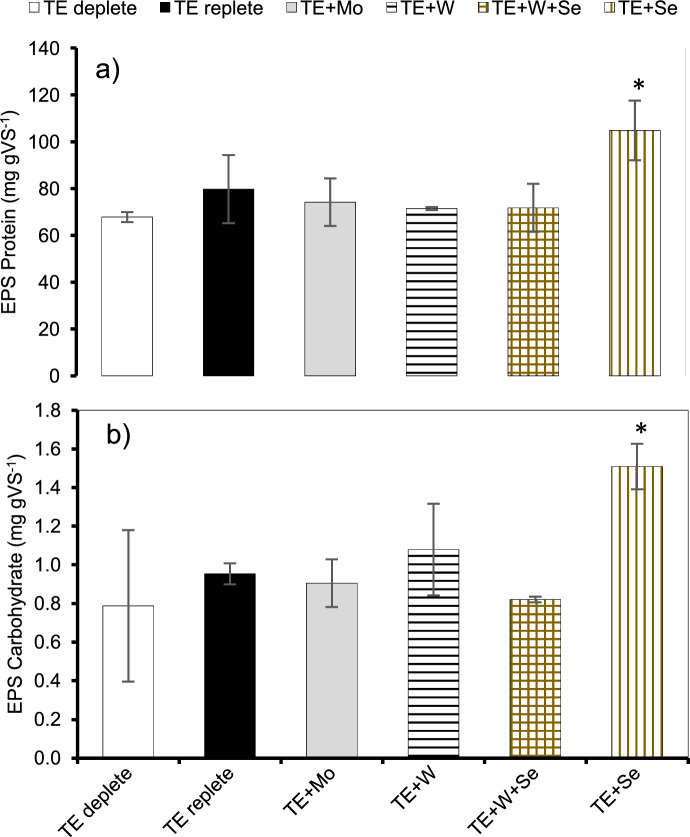
**a** EPS protein concentration and **b** EPS carbohydrate concentration after metal exposure (day 24). Fig Legend includes after metal exposure based on the specific groups (n = 3); (*) indicates statistically significant (p < 0.05) difference between the groups tested; TE + Se vs all groups

The co-occurrence of W + Se reduced EPS carbohydrate concentrations, when compared to TE-deplete and TE-replete samples (Fig. 6).

### Mo, W and W + Se increased relative abundance of fermentative taxa alongside metabolic potential for nucleotides and vitamins

The start-up period did not significantly affect the Shannon index and Simpson diversity metric, indicating consistent microbial diversity across the start-up phase. However, the presence of W + Se and Se group, led to reduction in richness and evenness of the community (Fig. S5). The dominant phyla were *Bacteriodota*, *Chloroflexota* and *Firmicutes* following W + Se, Se and Mo dosing. *Anaerolineaceae* family were dominant after metal dosing, specifically *Longilinea*, *Anaerolinea* and uncultured representatives. (Table 3 and Fig. S6).

**Table 3:** The relative abundances (RA) of phyla and taxa after takedown

Condition	Phyla relative abundance (%)	Bacterial Taxa relative abundance (%)	Archaeal Taxa relative abundance (%)
TE deplete	Chloroflexota (32), Firmicutes (18)	*Longilinea* (14.6), uncultured *Anaerolinea* (7.9), *Anaerolinea* (6.8), *Caproiciproducens* (10.8)	*Methanothrix* (9.2), *Methanobacterium (4.5)*
TE replete	Chloroflexota (34), Bacteriodota (16)	*Longilinea* (13.8), *Anaerolinea* (8.9), uncultured *Anaerolinea* (8.2), *Macellibacteroides* (10.2)	*Methanothrix* (8.9)
TE + Mo	Firmicutes (25) Chloroflexota (26),	*Caproiciproducens* (17.6), *Longilinea* (10.0), *TA06 phylum* (7.7), Other Bacteria (7.4)	*Methanothrix* (10.7), * Methanobacterium (4.8)*
TE + W	Chloroflexota (32), Bacteriodota (16)	*Macellibacteroides* (19.3), *Longilinea* (11.6), uncultured *Anaerolinea* (8.9), *Anaerolinea* (8.4), Other Bacteria (7.8)	*Methanothrix* (7.7)
TE + W + Se	Bacteriodota (41), Chloroflexota, (22–23)	*Macellibacteroides* (39.1–39.0), *Methanothrix* (9.7–7.5), *Longilinea* (8.6–10.3), *Anaerolinea* (6.4–5.4), uncultured *Anaerolinea* (4.8–5.3), *TA06 phylum* (5.6), Other Bacteria (5.7)	*Methanothrix* (9.7)
TE + Se			*Methanothrix* (7.5)

**Fig. 7:**
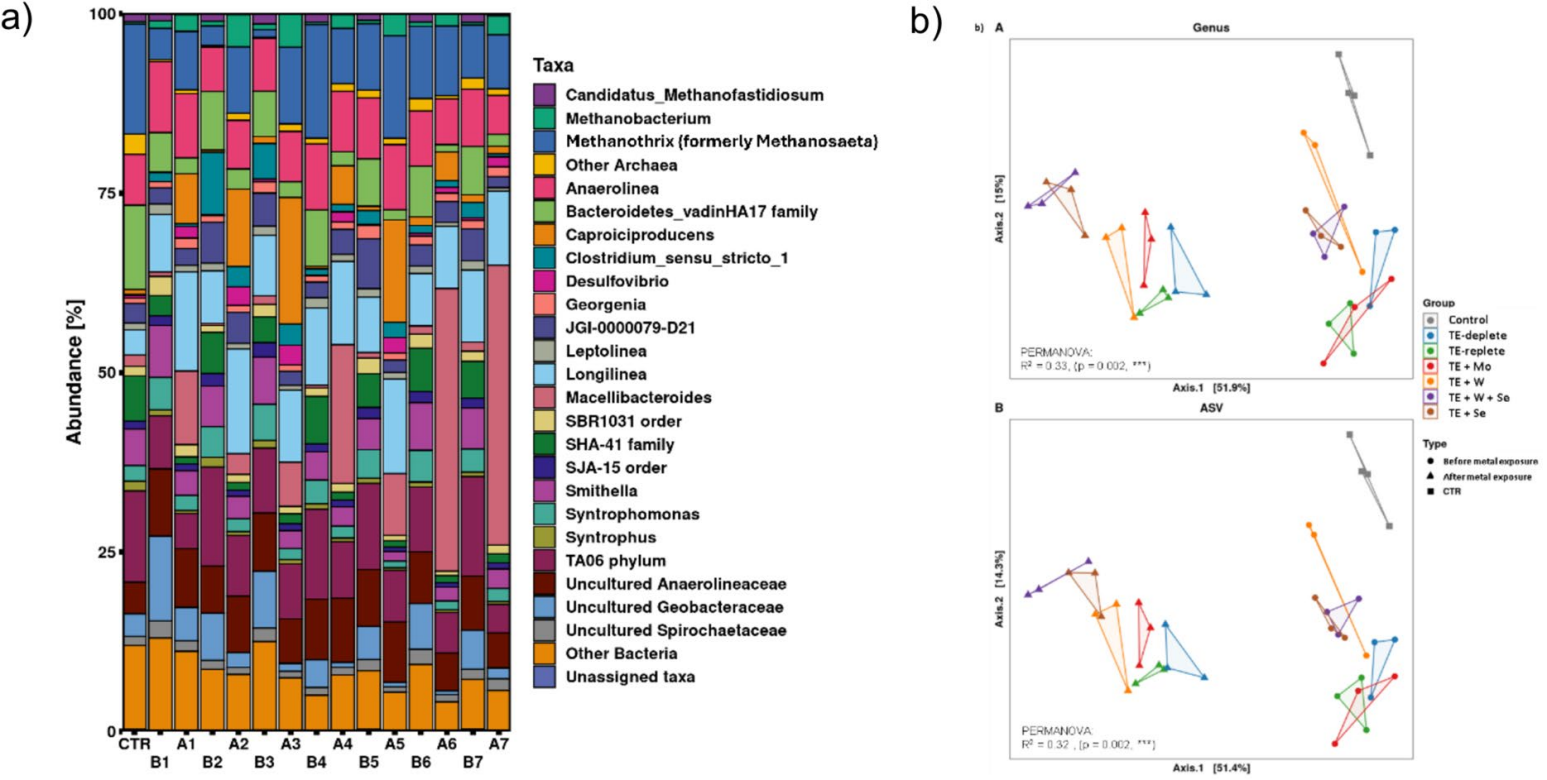
**a** Relative abundance plots of groups (B) Before metal exposure and (A) After metal exposure; Groups are represented as (1) TE deplete; (2) TE replete; (3) TE + Mo; (4) TE + W; (5) TE + W + Se; (6) TE + Se; (CTR) Inoculum **b** Principle component analysis of bray–curtis dissimilarity of A) Genus and B) ASV

Exposure to metals resulted in a dynamic shift in microbial community composition, which was explained by 51.4% variation between start-up and takedown, using a Bray Curtis metric, this statistical difference was shown by PERMANOVA (R^2^ = 0.32, p = 0.002). Clustering of samples before metal exposure (day 9), indicated community similarity. Metal addition effects were alike when TE + W, TE + Se and TE + W + Se were added, Se most impacted the clustering of these groups. Notably, TE replete, TE deplete and TE + Mo clustered together, indicating those have similar inter-group diversity (Fig. 7b).

**Fig. 8:**
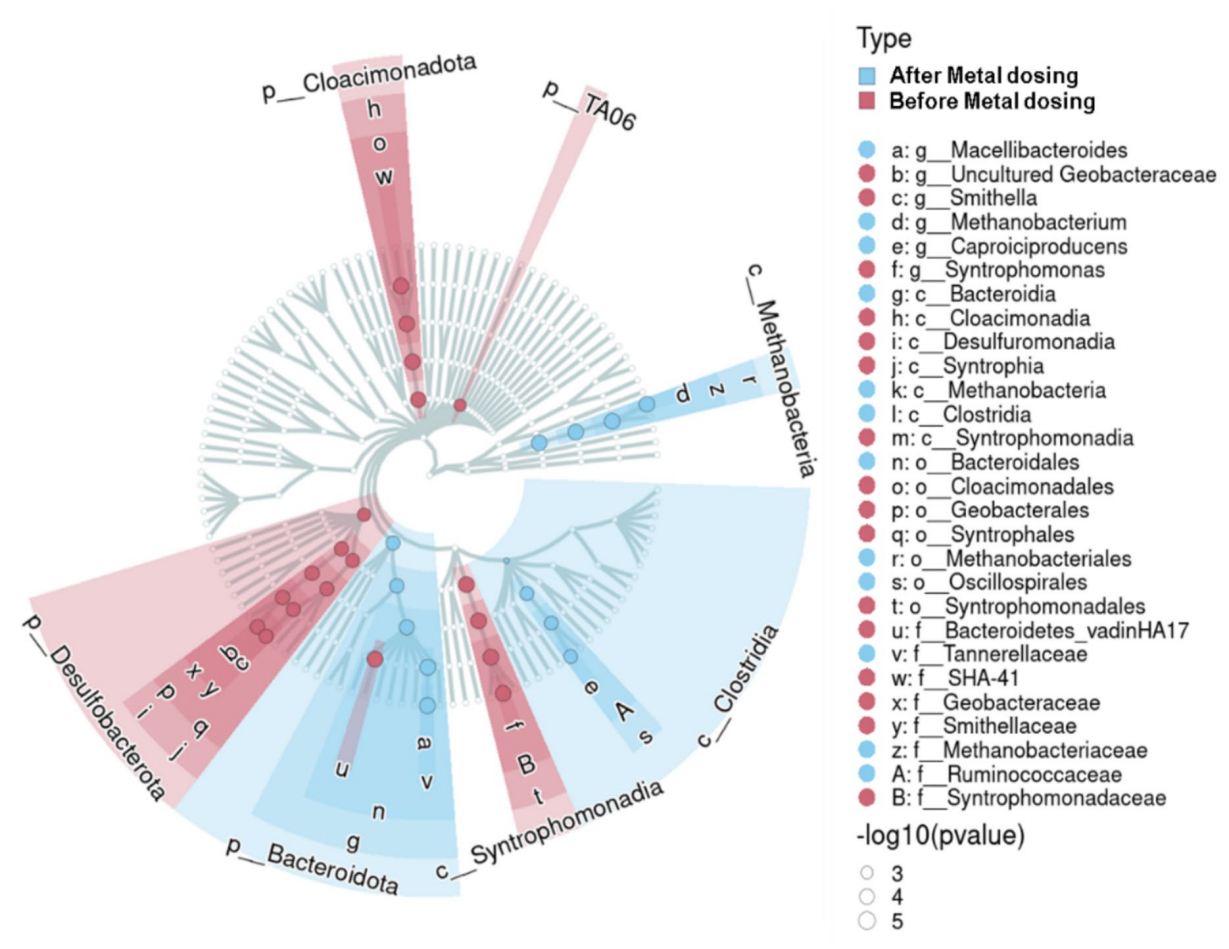
Least discriminant analysis effect size identifies critical ASVs of samples before metal exposure (red) and after metal exposure (blue). Strength of association is measured by the -log10 (p-value) indicated by the circle size representing higher associations with the Type. (Color figure online)

Least discriminant analyses identified dominant taxa within two groups. Before exposure, uncultured *Geobacteraceae, Syntrophomonas*, *Smithella*, *Bacteroidetes* vadinHA17, uncultured *Cloacimonadetes* and phylum *TA06* appeared to be differentially dominant across all the bioreactors (n = 6) (Fig. 8). After exposure, the fermenters were present, including *Macellibacteroides*, *Caproiciproducens, Longilinea, Methanobacterium* and *Clostridium *sensu stricto* 5* (Fig. 8 and S7).

The PICRUSt analysis revealed, Se exposure, compared to TE-deplete condition, led to an increased potential for purine biosynthesis. Mo exposure was associated with higher relative abundance (RA) of genes for pyrimidine metabolism, particularly to those related to adenosine and deoxyadenosine. Conversely, W + Se exposure resulted in guanosine and deoxyguanosine metabolism. Members of phyla *Chloroflexota* and *Bacteriodota* were major contributors of the cell wall (Fig. S9), carbon (Fig. S8) and nucleotide (Fig. S10) functional potential of the microbiome. Additionally, these taxa contributed to the biosynthesis and salvage pathways of tetrahydrofolate (Vitamin B9), pyridoxal (Vitamin B6), and cyanocobalamin (Vitamin B12) (Fig. S11).

The archaeal community was dominated by *Methanothrix (*formerly *Methanosaeta)*, maintaining an RA of ~ 10% across the experiments. *Methanobacterium* was only present in TE-replete (4.5%) and TE + Mo (4.6%) groups. *Candidatus_Methanofastidiosum* was only present in groups before metal exposure, and in the control incubations.

Lastly, exposure to specific metal groups led to a reduction in median number of 16S rRNA gene copies per gVS within the active bacterial community. The presence of TE + Se shows a significant reduction in the number of total transcripts (p < 0.01) than TE-deplete (p < 0.05). Mo addition had a negative correlation (-0.76) with 16S rRNA transcripts (p < 0.05) (Fig. S12).

## Discussion

### TE dosing is important for anaerobic digestion process

In AD system, the presence of Fe, Ni and Co is critical for maintaining stable biogas production from sulphur rich substrates (Gustavsson et al. 2013), high solids food waste (Kang et al. 2022) and overall AD process performance (Choong et.al., 2016). However, the independent roles of molybdenum (Mo), tungsten (W), and selenium (Se), particularly in combination with Fe, Ni, and Co, have not been extensively studied.

In this study, TE-replete and TE + Se conditions showed a significant improvement in methane production (Fig. 2), likely due to the low initial solid Se observed prior to metal exposure (Fig. 4b). Similar positive or neutral effects have been documented in biomass with a history of TE dosing or where no deficiency existed. For example, Facchin et al. (2013) reported increased methane production (45–65%) when Mo and Se were added to low-TE food waste, despite a high background TE substrate. The impact of Se on methane production has been confirmed by studies on both Se dosing (Feng et al. 2010) and deprivation (Šafarič et al. 2020; Worm et al. 2009).

This study adds to existing records around the role of Fe, Ni, Co, and Se dosing within methanogenic granules in fed batch mode, highlighting the importance of a start-up period to acclimatise biomass to synthetic feed, environmental conditions and shift in operation modes from expanded granular sludge bed (EGSB) to fed batch. Further studies should focus on demonstrating the W, Mo and Se depletion after stressed conditions (ammonia and inhibitory compounds), and TE dosing methods to recover AD microbiomes.

### Exposure to W + Se positively impacts acetoclastic methanogenesis

Based on the importance of trace metal dosing for methanogens (Scherer et al. 1983) this study demonstrated the presence of Se and the combination of W + Se increased acetoclastic methanogenesis significantly compared to TE-replete (p > 0.01) and TE + W (p > 0.05) (Table 2). Previous work with similar methanogenic granules, demonstrated, that indeed Se increases acetoclastic activity until concentrations of 500 µM Se, coupled with 90% Se removal up to 400 µM (Logan et al. 2003). In effect, demonstrating sludge Se tolerance and augmenting acetoclasts. The role of W in enhancing methanogenesis is supported by studies using waste sewage sludge, where W supplementation increased acetate consumption by acetoclastic methanogens, improving methane production by 2.17 times (Roy et al. 2022). Additionally, W and W + Se exhibited high CO_2_-reducing methanogenesis rates, suggesting a supplementary role in the AD process. While previous studies have shown that Ni and W can increase specific methane production (Das and Mondal 2013) and methanogen cell counts by 300%, shifting towards CO_2_-reducing methanogenesis (Arthur et al. 2022),

Further research is needed to clarify the function of long-term W pulse dosing on CO_2_-reducing methanogenesis, particularly in full-scale EGSB reactors, as well as techno-economic profile of W and Se dosing. Additionally, methylotrophic methanogenesis declined significantly due to the shift from full-scale EGSB to fed-batch operation or utilising synthetic feed (Keating et al. 2025) a critical factor that must be considered when setting up lab-scale reactors.

### Mo, W and Fe co-occurrence is essential for higher Fe/ S retention in anaerobic systems

Iron (Fe) is a crucial element in anaerobic digestion (AD) processes (Mary et al. 2024), playing key roles such as facilitating the formation of Fe-S clusters, which act as vital cofactors (Zhang et al. 2015), contributing to the modulation of sulfide concentrations, and the reduction of oxidation–reduction potential within reactors (Shi et al. 2011). Here, the co-occurrence of Fe with W or Mo in abiotic reactors led to a notable increase in Fe solubility. Subsequent solid metal analyses, suggests that more Fe was available for retention (Fig. 3a), thereby retaining more S (Fig. 4d), most likely from L-cysteine or feed components. It is established that Mo adsorbs effectively to FeS pyrites under anaerobic conditions at low pH (Xu et al. 2006), similar adsorption patterns have been documented for W (Cui and Johannesson 2017) and Se (Molaey et al. 2017). However, the enhancement of Fe solubility and S retention due to the presence of Mo or W has not been previously reported, making this original result an important driver of higher retention of other metals from the TE mix.

In anaerobic granular sludge, Fe-S minerals drive maximal sorption. This is particularly true for Co and Ni which show higher sorption in exchangeable and organic/sulfide fractions (van Hullebusch et al. 2005, 2006), with Cu (Cao et al. 2014) following a similar trend. Simultaneously, here, it is also likely the production of metal-thiol groups were involved in retaining intermediate metals (Fig. 3b, c and d), much like previously confirmed for Co and Ni (Yekta et al. 2014) in AD. Such improved retention is vital for enzymes like mcrA, essential for methanogenesis (Zhang et al. 2009). Previously, the co-occurrence of Co and Fe lead to higher retention levels in paper mill AD sludge (Osuna et al. 2004). These findings collectively suggest that Fe & S likely drive TE dynamics in AD. Our study corroborates this idea, based on retention trends of Co, Ni, and S (Fig. 3b, c and d).

In this study, baseline removal of Ni and Cu from TE-deplete group indicated that feed substrates and macronutrients may contain ultra-trace concentrations of these metals, which likely accumulated over the 24-day trial. Thus, suggesting an active procurement of Ni and Cu from the environment. Typical of the Ni, Co dependent utilisation and uptake capacity shown via *in-silico* genomes and proteomes of *Chloroflexota*, Deltaproteobacteria and Methanosarcinales (Zhang et al. 2009).

To the best of our knowledge, this is the first study to experimentally demonstrate that simultaneous dosing of Mo and W significantly impacts Fe/S retention in methanogenic systems.

### Se drives EPS generation in methanogenic granules

In this study, we observed an increase in Se precipitate stimulates EPS protein and carbohydrate levels. The presence of Se^0^ in anaerobic granular sludge from brewery wastewater led to its complete accumulation within and surface of biomass. This accumulation largely identified as periplasmic cytochrome proteins and lipids (Gonzalez-Gil et al. 2016) backed the stability of colloidal Se nanoparticles (Jain et al. 2015) too. Se potentially incited such a stress buffering response here as well that could underpin improved methanogenic performance based on positive correlation (r^2^ = 0.87, p < 0.05) between total Se and specific methanogenic activity against acetate (Table 2). It must be noted that, previous studies have revealed that short air exposure to Se species caused higher accumulation of Se in the organic and residual fraction during sequential extraction (Lenz et al. 2008). The sludge in this study was exposed to ambient air during the Decant-Fill stage of the experiment and possibly played a part in Se retention as well.

Nevertheless, both physico-chemical and microbial processes contribute to reduction of Se in methanogenic granules (Astratinei et al. 2006). The reduction products of SeO_3_^−^ may induce toxicity (Lenz et al. 2006) and pinpointing their exact source is complex (Tan et al. 2016). Therefore, further research on the microbial players, genomic features, expression of Se EPS capping proteins and impacts on methanogen functional capacity is important. Delineating these mechanisms will be important for high-treatment capacity sludge.

### Fermentative taxa predominate the microbiome after Mo, W and Se dosing

The microbial community in anaerobic granular sludge exhibited significant shifts following the administration of Mo, W, and Se, predominated by fermentative taxa such as *Anaerolineacaea* (*Longilinea* and *Anaerolinea*). These syntrophic fermenters were previously associated with *Methanothrix* to increase the acetate pool for methane production from alkanes (Liang et al. 2015) and acid hydrolysates from *Agave tequilana* var. *azul* bagasse (Snell‐Castro et al. 2019). Fluorescence staining suggests that *Anaerolinacaea* were co-located with *Methanothrix* in AD sludge (McIlroy et al. 2017). Thus, strengthened synergy between fermentative taxa and methanogens during metal addition was evident in our study (Table 3 and Fig. 7a). Certain fermenters were observed as potentially valuable indicator species. For instance, *Macellibacteroides* ASV, identified as an isolate from an upflow anaerobic filter (Jabari et al. 2012), is recognized for its resilience in extreme environments. The relative abundance (RA) of this ASV increased under the TE + Se and TE + W + Se conditions, suggesting its presence is Se-dependent, as no similar change was observed in the TE + W group (Fig. 7a). Therefore, this taxon may serve as an indicator for excess Se in methanogenic granules.

**Fig. 9:**
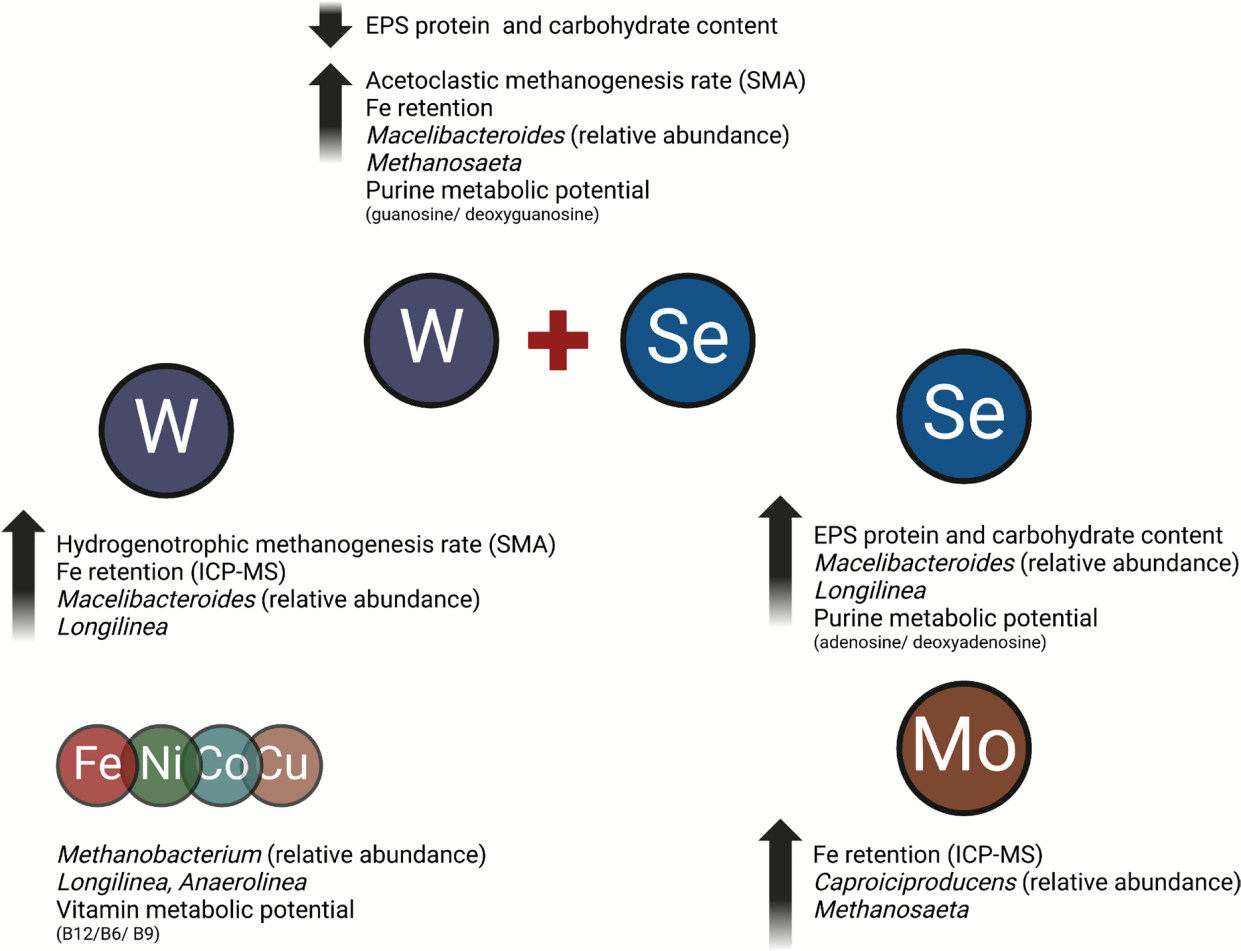
Summary of the results in this study and potential associations and trends. This image was created in Biorender

*Capriciproducens* species demonstrated an increased RA in TE + Mo. This taxon has a role in low pH environments (~ 5.0) (Zhang et al. 2022) and was suggested as an indicator of anaerobic co-digestion of sludge and wheat straw (Kang et al. 2021) and responsible for chain elongation from galactitol to caproic acid (Kim et al. 2015) and in mixed acid production (Flaiz et al. 2020). Similarly, *Clostridium *sensu stricto* 5* appeared to be influenced by the presence of W and Mo, as evidenced by an increased RA compared to TE-replete and TE-deplete groups. (Fig. 9).

The competitive advantage of fermentative taxa in response to metal dosing is their inherent functional redundancy. This adaptability allows them to thrive under dynamic conditions, such as acidosis (Bertucci et al. 2019), chain elongation processes (Liu et al. 2023) and here, likely enhance their tolerance to TE variations. This shift is consistent with the reduced RA in syntrophic bacteria after metal exposure (Fig. 8), as metabolically versatile taxa became dominant, corroborating earlier findings (Werner et al. 2011). Simultaneously, the absence of Candidatus_*Methanofastidiousum* after metal exposure, may be related to the suppression of methylotrophic methanogenesis, particularly in the context of methanol utilization rate (Table 2) and presence/ absence in group conditions.

Nucleotide metabolism is essential for the survival and growth. In this study, the presence of Mo and W + Se increased RA of pyrimidine biosynthesis potential. This is necessary for the replication, recombination and repair function in AD microbiomes, particularly under ammonium stress (Li et al. 2017) and, ensuring nucleotides are available for cellular growth (Lohani and Havukainen 2017).

Vitamin sharing for communal benefit is a crucial factor in shaping the structure and function of the human skin (Swaney et al. 2022) and gut (Ortiz et al. 2022) microbiome. Within the gut, for instance, commensals produce and share vitamin B9, promoting community stability and structure (Soto-Martin et al. 2020; Engevik et al. 2019). While auxotrophy has been reported among acidogens (Sjostrom et al. 1953) and methanogens (Buchenau and Thauer 2004). The full extent of folate metabolic potential within AD systems remains elusive. This study suggests the likely presence of vitamin B9 and B12 metabolic potential (Fig. S12), under TE-replete, W + Se, and Se dosing, with putative positive contribution from genera *Anaerolinea* and *Macellibacteroides* (Fig. S11). Previous research highlight’s role of vitamin B12 in AD; vitamin B12 as opposed to CoCl_2_ dosing, can significantly enhance methane production (3x) (Fermoso et al. 2010), and its absence is detrimental to methanogenesis (Kenealy and Zeikus 1981). Additionally, the supplementation of metals such as Mo, B, Cu, Zn, Co, Fe, Ni, and Mn has been shown to stimulate both vitamin B12 production and methane generation in AD processes, offering a favourable benefit–cost ratio (Ariunbaatar et al. 2016; Izadi et al. 2020). The crucial role of B vitamins, particularly in response to strategic metal dosing, demands further investigation, specifically measuring intra and extracellular vitamin concentrations during dosing scenarios, thus investigating public goods sharing in methanogenic granules. Unlocking these dynamics will be key to develop robust and efficient AD systems.

Ultimately, optimising metal dosing strategies for future full-scale reactor operations is important. Operators should tailor these strategies to specific characteristics and background TE levels of their sludge and feedstock. Emphasis should be on the addition of Fe, Ni, and Co, with careful consideration to their mix composition and concentration. Dosing times must be adjusted to achieve specific goals, such as higher retention and EPS production by adding Se, or enhanced methanogen performance by adding W + Se. These strategies need to be demonstrated in lab-scale and pilot scale reactors, prior to scale up. Furthermore, adopting optimal, model-driven metal dosing regimens, like selecting metal chlorides and adhering to a low-frequency dosing schedule (George et al. 2024), will be crucial for success in AD operations.

## Conclusions

This study demonstrated that co-dosing methanogenic granules with Mo, W, and W + Se alongside a trace metal mix, induced distinct shifts in extracellular polymeric substances (EPS) concentrations, microbiome composition, and functional potential. In particular, W + Se and Se imposed positive effect on methane production via significantly stimulating acetoclastic methanogenesis, while Se increased EPS protein and carbohydrate contents. These effects were associated with higher Fe solubility and enhanced Fe and S retention in the presence of Mo and W as confirmed by SEM–EDX. This differential retention of metals coincided with a higher RA of fermentative taxa such as *Anaerolineaceae*, *Macellibacteroides* and *Caproiciproducens* sp., alongside lower total bacterial transcript levels. Functional analysis revealed higher abundances of nucleotide metabolism, but most importantly Vitamin (B9, B12, B6) potential which suggests the important capacity of metal stimulating vitamin precursors within methanogenic consortia. These findings highlight that tailored combinations of trace metals could modulate both process performance and microbiome function, providing a strong basis for optimising anaerobic digestion operations both at batch scale. Further demonstration of these combinations at lab-scale and pilot EGSB reactors will facilitate their industrial applications and prove utility for AD operations.

## Supplementary Information

Below is the link to the electronic supplementary material.

Supplementary file1 (DOCX 1569 KB)

## Data Availability

The raw 16S rRNA amplicon sequencing data was submitted to the SRA (Sequence Read Archive) under BioProject number PRJNA1290086.
