# Supplementary material for: Mutual dosing of tungsten, molybdenum and selenium impact anaerobic digestion microbiome
Source: Biometals. 2026 Jan 28;39(2):565–86. doi: 10.1007/s10534-026-00790-1 (PMC13083482; doi:10.1007/s10534-026-00790-1)
Supplement: Supplementary file 1 — Supplementary file1 (DOCX 1569 KB) [file 10534_2026_790_MOESM1_ESM.docx]

# **Supplementary section**

**Mutual effects of tungsten, molybdenum and selenium on granular methanogenic consortia**

**Kris Anthony Silveira^1, 2,3^, Javier Ramiro-Garcia^2^, Cian Lawless^3^, Jose Manuel Espinosa-Vazquez^2^, Fernando Gonzalez-Fermoso^2^, Gavin Collins^3^, Vincent O’Flaherty^1^**

1- Microbial Ecology Laboratory, School of Biological and Chemical Sciences, University of Galway, University Road, Galway, H91 TK33, Ireland.

2- Bioprocess for the Circular Economy Group, Instituto de la Grasa, Consejo Superior de Investigaciones Cientificas (CSIC), Campus Universitario Pablo de Olavide-Ed. 46, Ctra. De Utrera, km 1, Seville 41013, Spain.

3 – Microbial Communities Laboratory, School of Biological and Chemical Sciences, University of Galway, University Road, Galway, H91 TK33, Ireland.

Mail addresses:

Corresponding author: [kris.silveira13@gmail.com](mailto:kris.silveira13@gmail.com); [vincent.oflaherty@universityofgalway.ie](mailto:vincent.oflaherty@universityofgalway.ie)

**Fig. S1** ml methane production per time point per batch for each condition.

**Fig.** **S2** tCOD removal per batch per condition

**Fig.** **S3** Biogas composition end of each batch per conditions.

**Fig.** **S4** Soluble metal removal in anaerobic bioreactor effluent from Decant stage across day 12; batch 4, day 18; batch 6 and day 24; batch 8 quantified as per Methods Section, Soluble metal quantification a) Iron, b) Cobalt, c) Nickel, and d) Copper. Standard deviation for triplicate measure soluble metal effluent concentration.


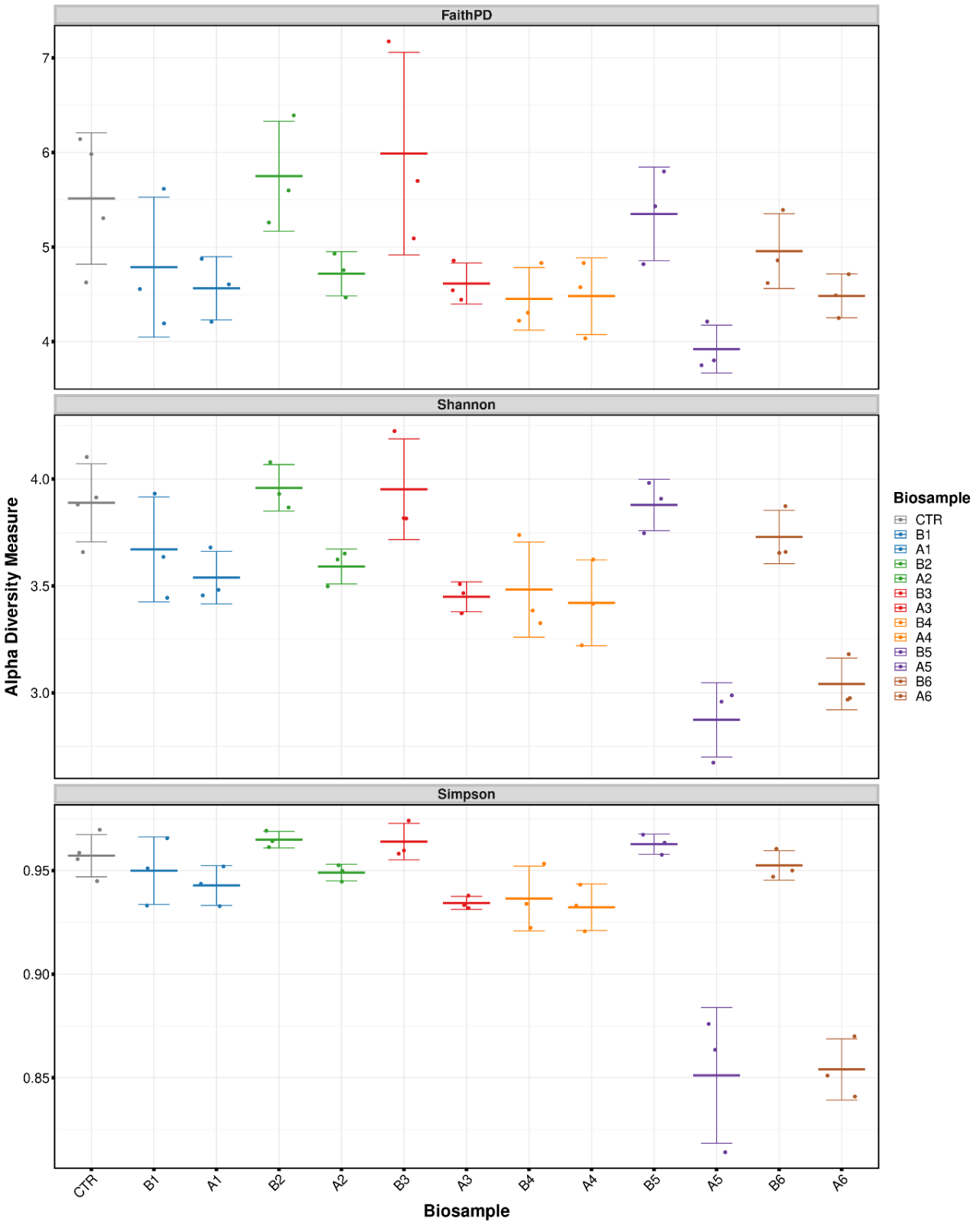


**Fig.** **S5** Measure of mean species diversity within local test group i.e. Alpha diversity. Three main metric employed a) Faith PD (includes phylogenetic distance by branch length of the species within the group), b) Shannon Index and c) Simpson Diversity (1-D) (include count and proportionality of species present within a group). Figure Legend include all groups, **Before (A)** **and After (B)** metal exposure based on the specific groups (n=3) as such : (1) TE deplete; (2)TE replete; (3)TE Mix + molybdenum; (4) TE mix + tungsten; (5) TE mix + tungsten+ selenium; (6)TE mix + selenium; (CTR)Control/ Raw inoculum .


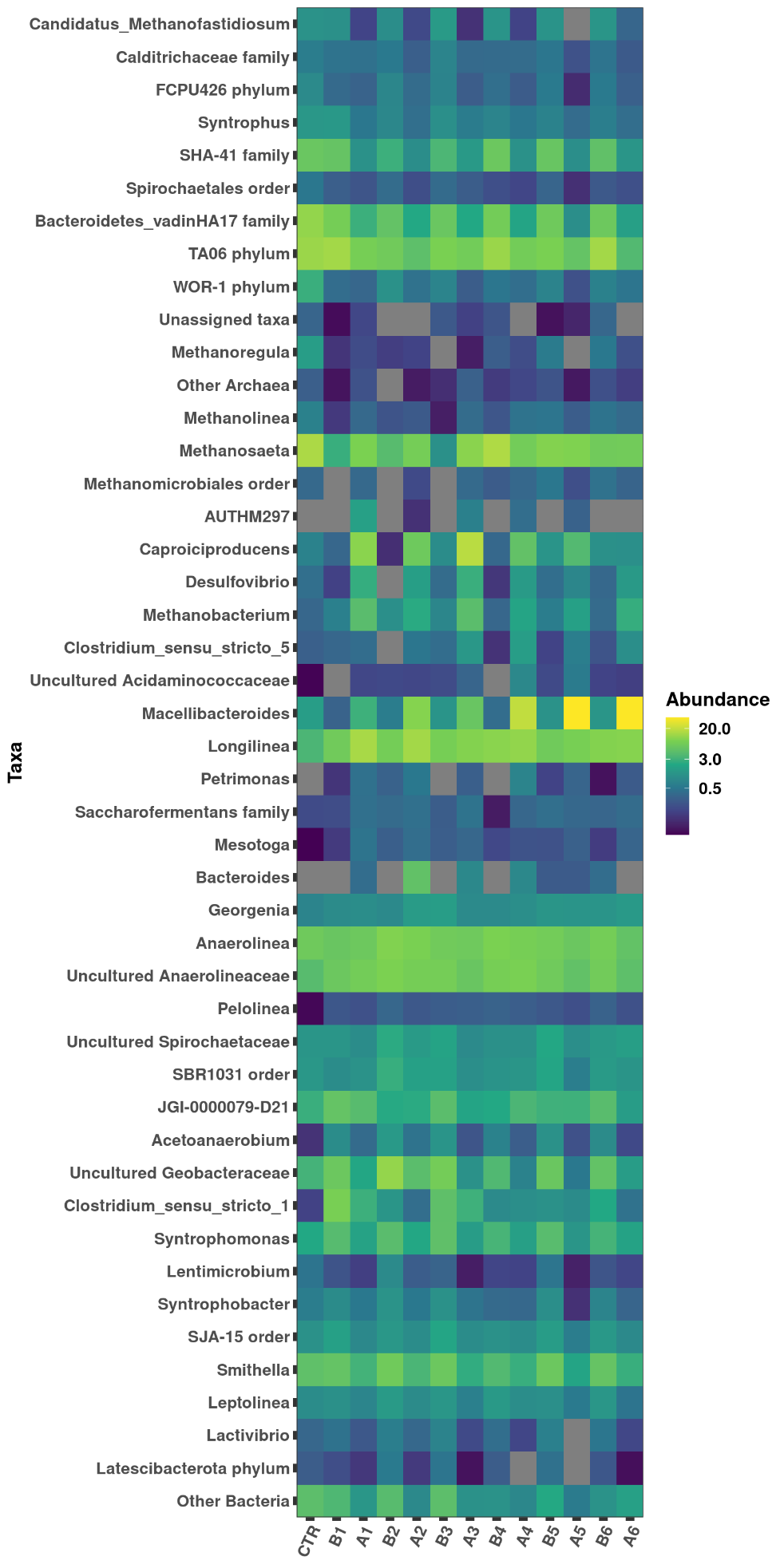


**Fig.** **S6** Heat map of relative abundance values based on two treatment groups. Comparison drawn between groups, B Before metal exposure; A After metal exposure include specific groups (n=3) as such: (1) TE deplete; (2)TE replete; (3)TE Mix + molybdenum; (4) TE mix + tungsten; (5) TE mix + tungsten+ selenium; (6)TE mix + selenium; (CTR)Control/ Raw inoculum. Taxa names are listed on the x –axis. Treatment group cells receive colour scales based on relative abundance value, yellow (high relative abundance score), blue (low relative abundance score) and, grey (not detected)


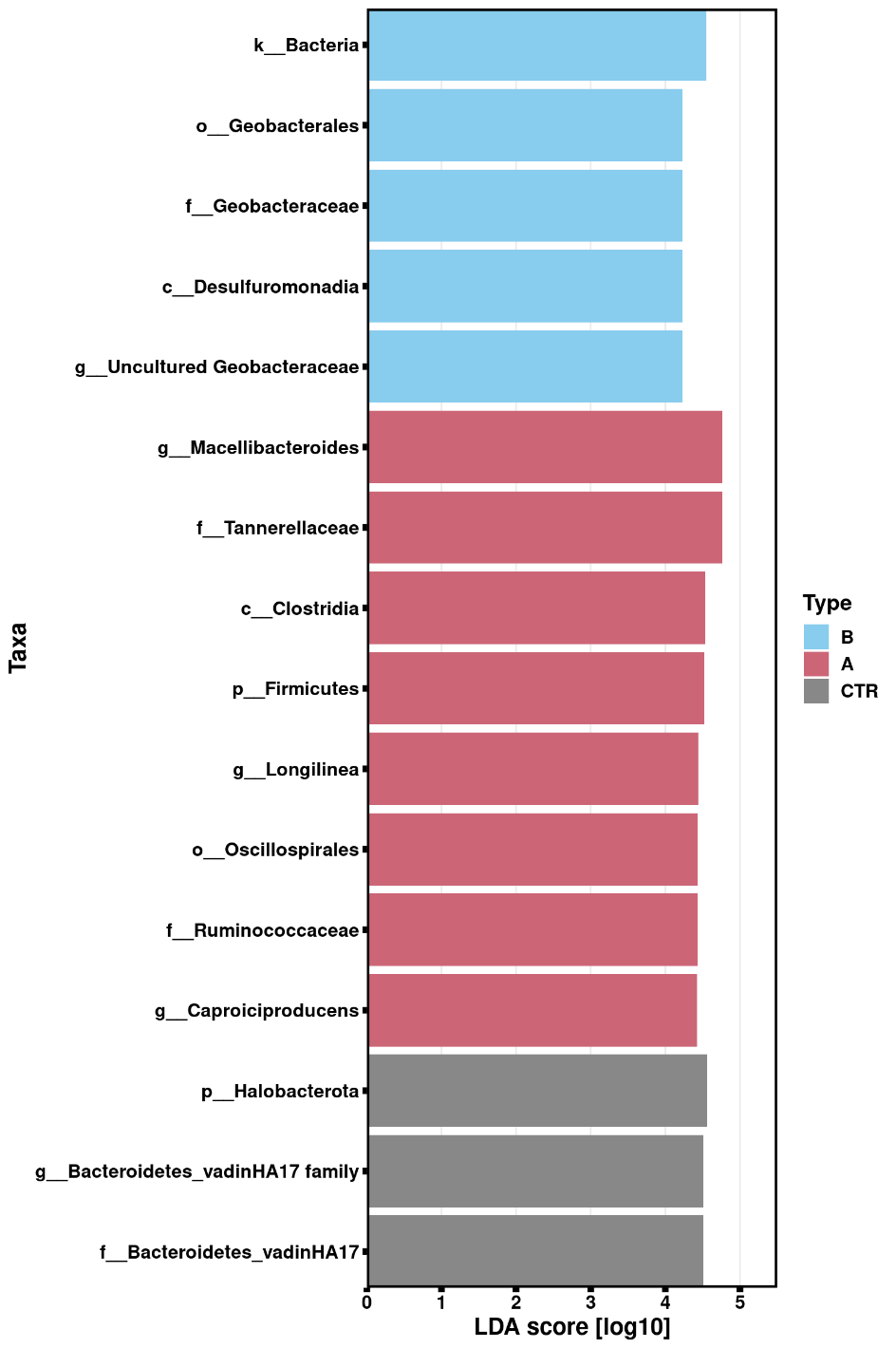


**Fig.** **S7** Log 10 Least Discriminant Analysis score for before and after metal exposure treatments.


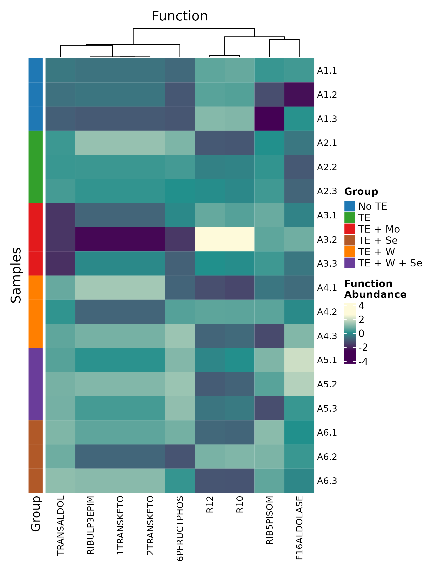

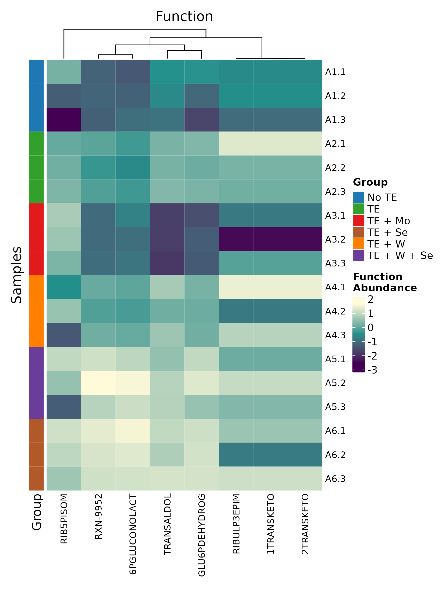

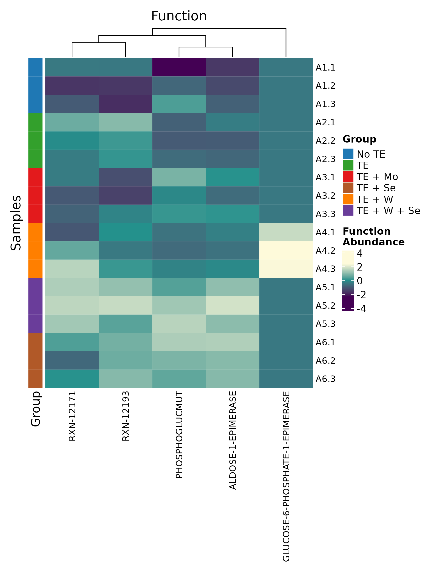


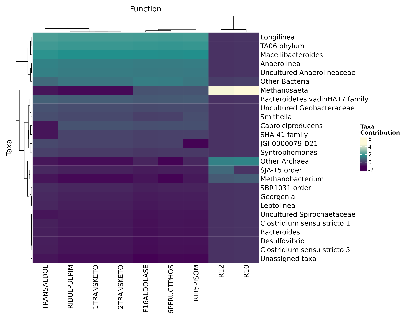

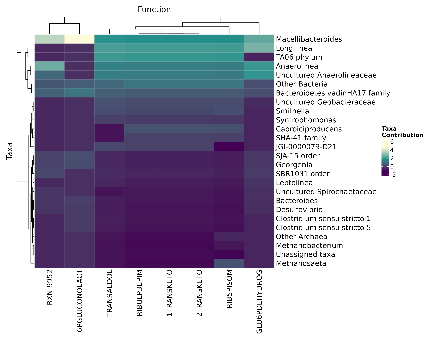

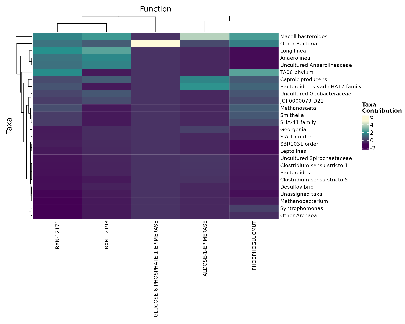


**Fig.** **S8** Carbon metabolism gene relative abundances and taxa contribution to the relative abundances of this pathway.


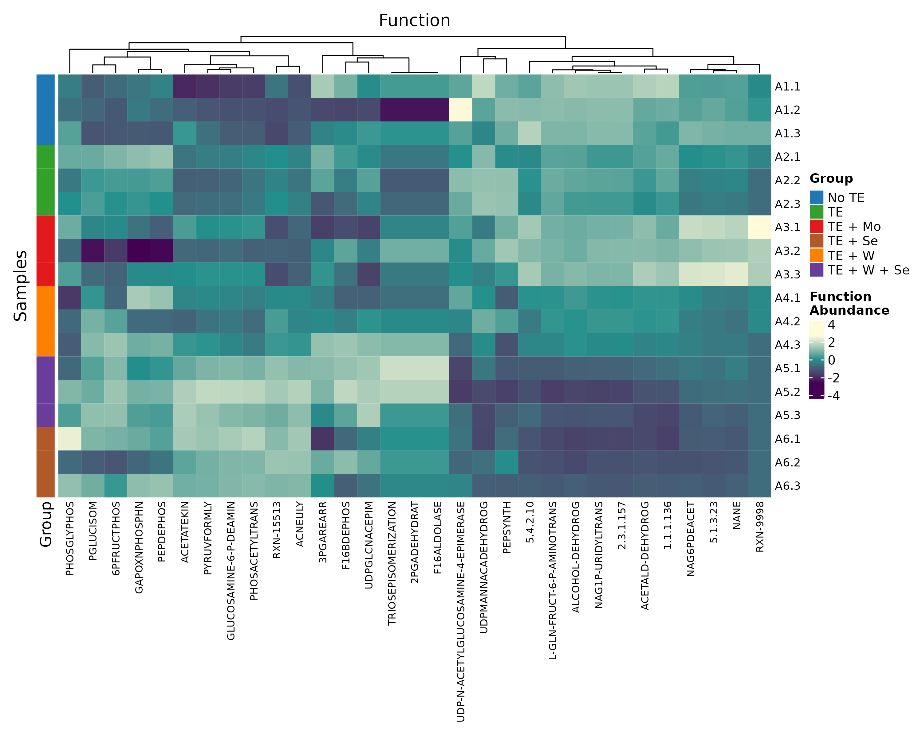

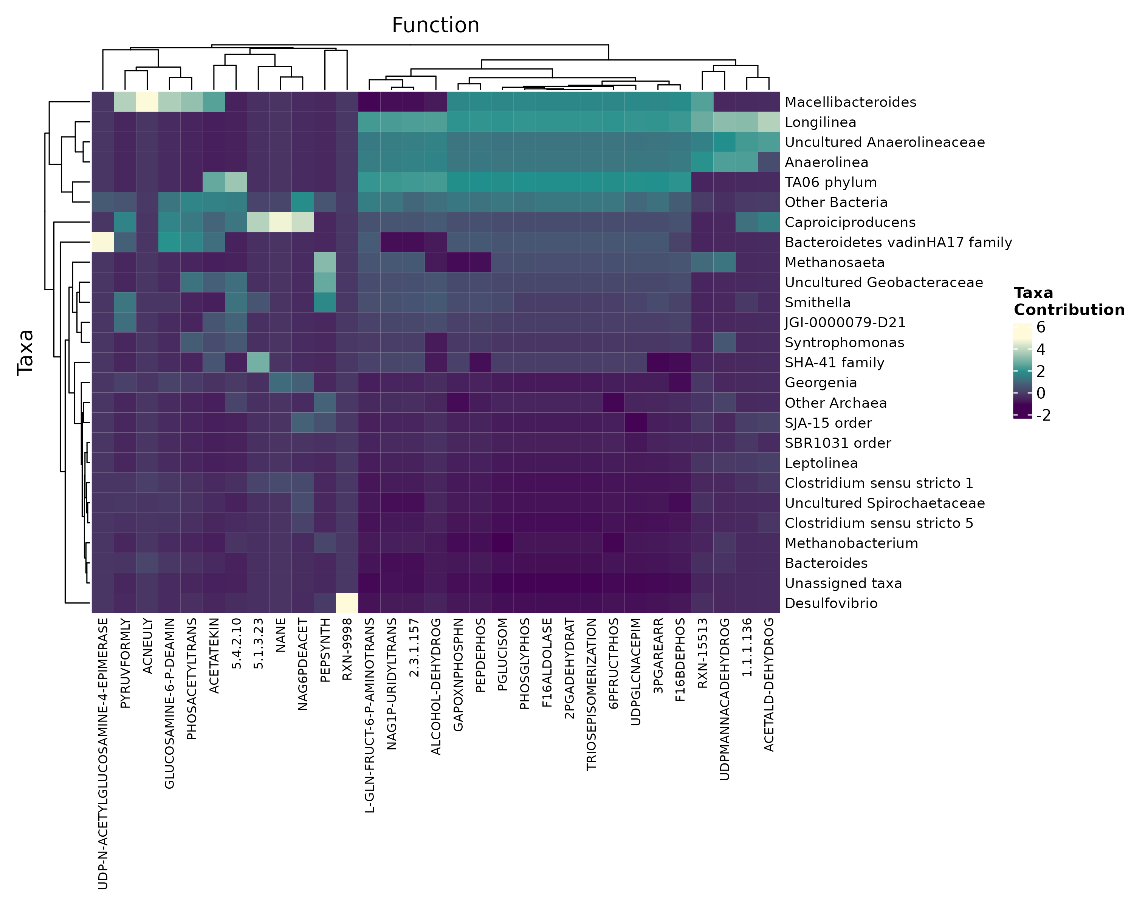


**Fig.** **S9** Cell wall metabolism gene relative abundances and taxa contribution to the relative abundances of this pathway.


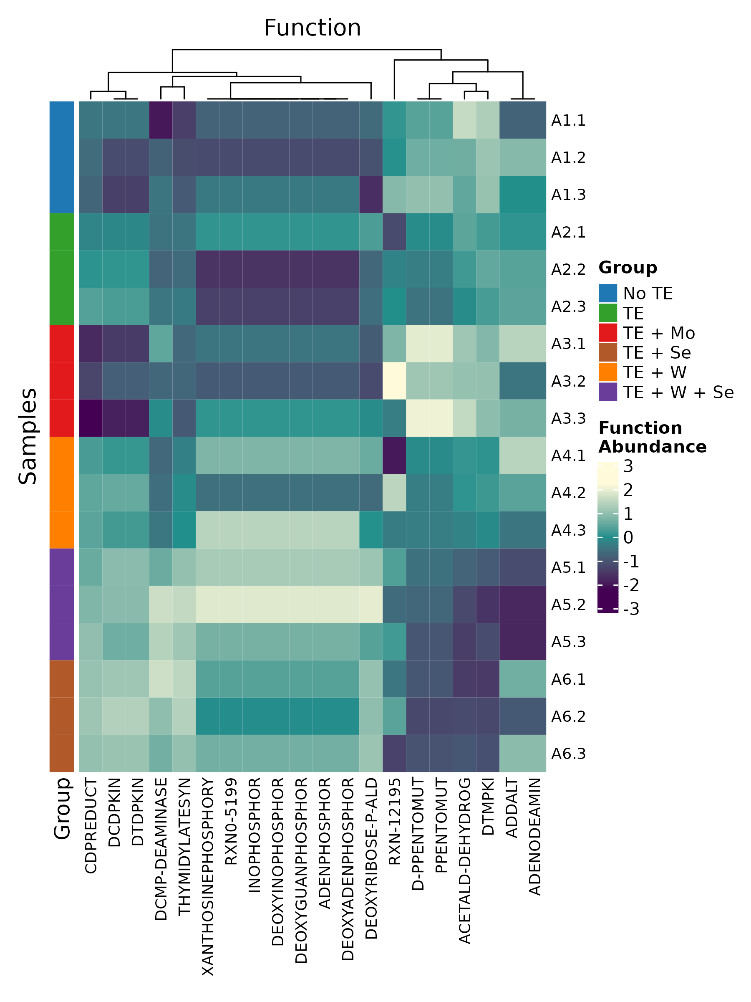

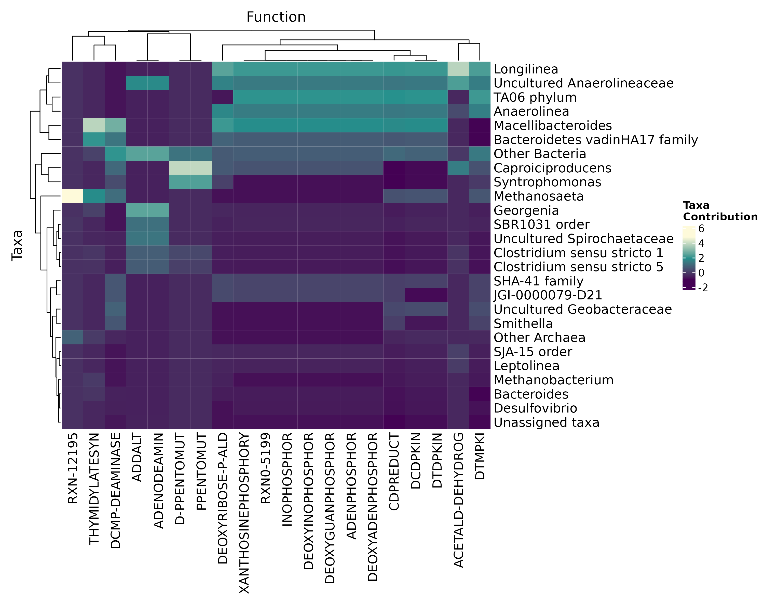


**Fig.** **S10** Purin and Pyrimidine metabolism gene relative abundances and taxa contribution to the relative abundances of this pathway.


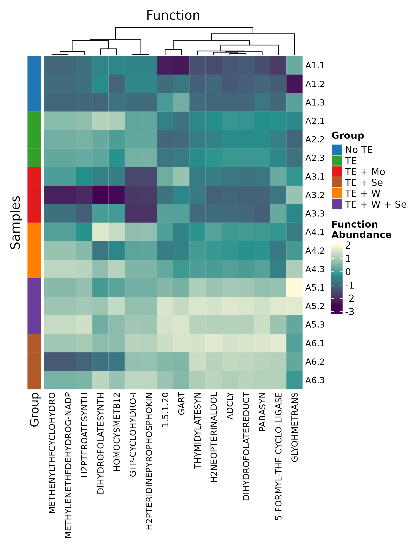

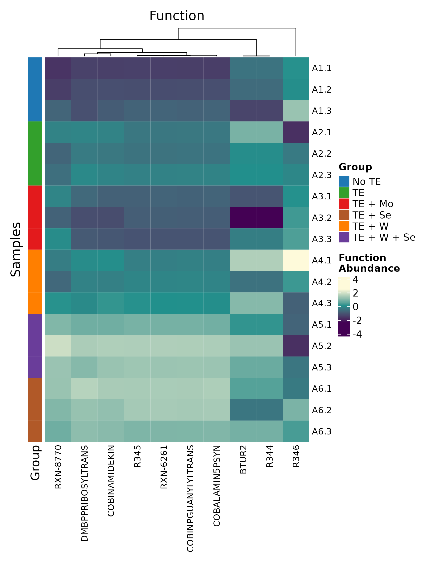

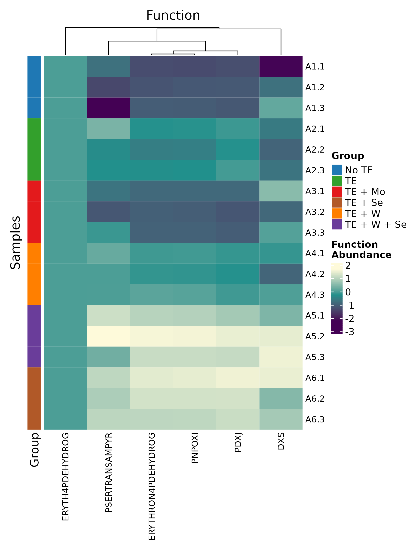


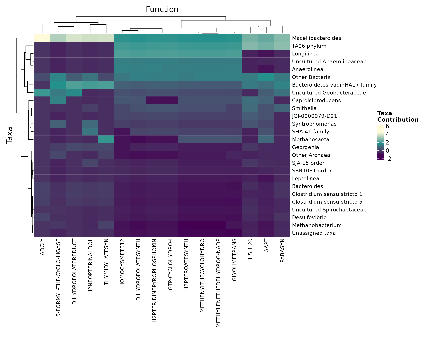

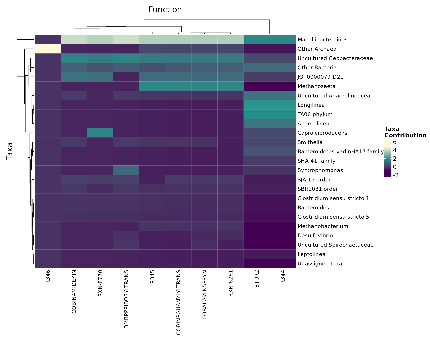

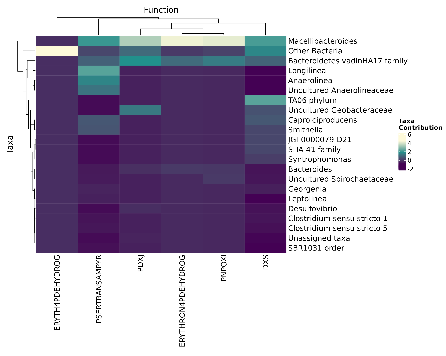


**Fig.** **S11** Vitamin B6, B9 and B12 metabolism gene relative abundances and taxa contribution to the relative abundances of this pathway.

*

**

**Fig. S12.** a) Quantification of 16SrRNA transcripts at startup (batch 3; day 9) (n=3) and takedown (batch 8; day 24) (n=18). Takedown conditions is shown in striped colours, Takedown condition is in solid colours; Median values ± Standard deviations plotted as error bars.

* - indicates statistically significant difference p <0.05, between before and after TE-deplete

** - indicates statistically significant difference p value <0.01, between before and after TE+Se
